# Implementing a Weekend Inpatient Robotics Program Using the Plan-Do-Study-Act Method: A Quality Improvement Initiative by Occupational Therapy Students

**DOI:** 10.7759/cureus.109367

**Published:** 2026-05-21

**Authors:** Trent Maruyama, Michelle Ashley, Lauralei Dirks, Rebekah Givens, Gwen Mohr, Cynthia Sloan, Delaney Wright, Sara J Stephenson

**Affiliations:** 1 Department of Rehabilitation Services, Neuro-Rehabilitation Center, Barrow Neurological Institute, Phoenix, USA; 2 Department of Occupational Therapy, Northern Arizona University, Phoenix, USA

**Keywords:** occupational therapy, quality improvement, rehabilitation, robotics, weekend therapy

## Abstract

Purpose

This quality improvement project assessed the feasibility, safety, and patient acceptability of a student-assisted weekend robotics program in an inpatient rehabilitation facility (IRF), with an exploratory analysis of functional outcomes. Occupational therapists in an inpatient rehabilitation unit created and assessed a sustainable weekend robotics program for patients.

Materials and methods

The inaugural weekend program was developed using a quality improvement framework, the Plan-Do-Study-Act (PDSA) method, which aligns with the knowledge translation framework. A small, random sample of patient data was assessed to determine the efficacy of the program and the utility of the measurements for future cycles.

Results

Over 12 weeks, 55 patients received 120 weekend robotic sessions and showed improved shoulder flexion and high satisfaction (98% positive). Patient diagnoses included neurologic, pulmonary, and orthopedic conditions. Detailed errors in the data were identified, and these supported the quality improvement cycle as researchers learned from the issues identified.

Conclusion

The successes of the program included its sustainability and approval for further PDSA cycles, suggesting that weekend robotics can enhance therapy dosage and patient engagement. The failures involved methodology issues with measurements, data collection and consistency, and student and therapist training.

## Introduction

Inpatient rehabilitation facilities (IRFs) must provide at least three hours of therapy, five days per week, under the Centers for Medicare and Medicaid Services (CMS) guidelines. However, resource constraints, such as staffing and reimbursement limitations, may prevent expansion beyond the CMS minimums [1]. Despite this situation, patients express a desire for additional therapy services, and evidence suggests that increased therapy dosages can enhance functional independence, quality of life, and recovery speed [2-5]. Institutional barriers, including fixed staffing models and no billing codes for advanced technologies like robotics, further limit opportunities to meet this patient-driven demand [1,6]. Addressing these challenges requires innovative, cost-effective solutions that optimize resources while improving patient outcomes.

Advancements in robotic technology have positioned it as a valuable tool in rehabilitation, enhancing recovery through high-intensity, repetitive, and task-specific training [7,8]. Upper extremity robotic devices optimize neuroplasticity by increasing therapy dosage and repetitions, potentially leading to cortical reorganization and improved functional outcomes, such as strength and dexterity [9-11]. The 2016 American Heart Association and American Stroke Association guidelines endorse robotic therapy alongside traditional methods to improve upper extremity function in stroke survivors, particularly in the subacute and chronic phases, when delivered by trained professionals [12,13]. Robotics can reduce limitations from conditions like hemiparesis, supporting its integration into IRF programs to boost patient independence [2,5,14]. Given the evidentiary support of robotics in IRF programs, it is important for programs to consider how to take this evidence and apply it to practice.

Knowledge translation bridges research and evidence-based practice in occupational therapy (OT), often using quality improvement frameworks like Plan-Do-Study-Act (PDSA) to implement novel care models [15,16]. PDSA, widely adopted in healthcare quality improvement, supports iterative testing of small-scale interventions, making it ideal for pilot testing sustainable programs [17]. This project applied knowledge translation through PDSA to test a pilot student weekend robotics program at an IRF. Like PDSA, knowledge translation describes a systematic and repetitive process of disseminating knowledge to end users for practical application [16,17]. Integrating an upper extremity robotic device to promote neuroplasticity and motor recovery through high-intensity, repetitive training [13,14,18,19], OT students intended to increase therapy access without incurring additional staffing costs. Practicing clinicians interested in participating in research rarely have the time or support to engage in research projects. By applying knowledge transfer principles in the PDSA process and reflecting on successes and lessons learned, we aim to contribute to the growing body of translational research in OT.

Internal patient satisfaction surveys at Barrow Neurological Institute’s Neuro-Rehabilitation Center revealed a desire for weekend therapy and robotic interventions, which enhance engagement through gamification [20]. This patient care quality-improvement project leveraged these insights to design a supplemental program and assessed its feasibility and impact on patient outcomes (e.g., shoulder flexion) and satisfaction. By challenging the traditional 1:1 care model, we sought to demonstrate how student-clinician partnerships and technology can address unmet needs in IRF settings, offering a replicable quality improvement strategy (PDSA) for broader healthcare applications. This project highlights the successes and challenges of one program’s engagement in research by implementing an evidence-based weekend robotics program. The objective of this quality improvement project was to assess the feasibility, safety, and patient acceptability of implementing a student-assisted weekend robotics program in an IRF, with an exploratory analysis of functional outcomes. While evidence exists for weekend therapy and robotic rehabilitation separately, the integration of these approaches through a student-assisted delivery model, within a PDSA quality improvement framework, represents a novel implementation that has not been previously evaluated.

## Materials and methods

Program design

**Figure 1:**
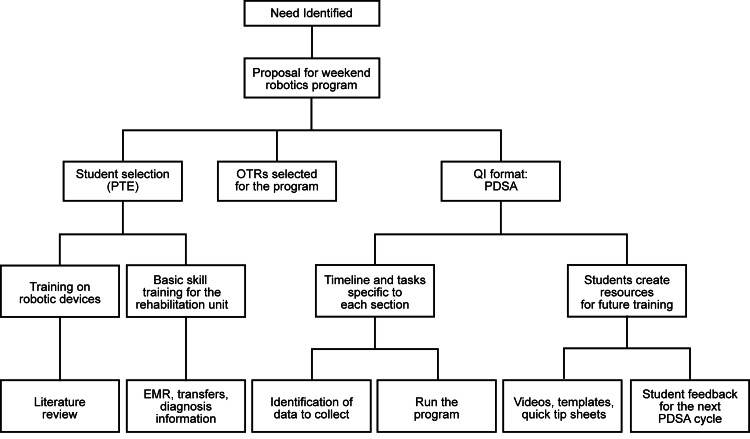
The research team’s planning process Credit: Used with permission from Barrow Neurological Institute, Phoenix, Arizona. EMR, electronic medical record; OTR, registered occupational therapist; PDSA, Plan-Do-Study-Act; PTE, Pi Theta Epsilon; QI, quality improvement

This quality improvement project was a collaboration between the Occupational Therapy Department at Northern Arizona University (NAU), Phoenix, AZ, USA, and the Department of Rehabilitation Services at Barrow Neurological Institute’s Neuro-Rehabilitation Center, an IRF in Phoenix, AZ, USA (Figure 1).

Five second-year OT doctoral students from NAU’s Pi Theta Epsilon honor society partnered with IRF staff to pilot a weekend robotics program. Student training in robotics and therapy delivery leverages the initiative of supplemented standard care without increasing staff costs. Because this study describes a quality improvement project, institutional review board approval and patient consent were not required.

Measurements

Four outcome measures assessed program efficacy and feasibility: (1) shoulder flexion active range of motion (functional improvement, measured via goniometer [21]), which was selected as an accessible impairment-level outcome measure for this first PDSA cycle due to its ease of measurement and relevance to upper extremity robotics interventions; (2) heart rate and oxygen saturation (SpO₂) (safety, monitored via standard devices); (3) Borg Rating of Perceived Exertion Scale (patient effort [22]); and (4) mood (satisfaction, measured on a visual analog scale [23]). Data were recorded in the IRF’s electronic medical record system and transferred to a cloud-based spreadsheet. For this pilot program, a random sample of 10 complete data sets was analyzed to evaluate the utility and impact of the program. At the end of each session, the OT students asked the patients whether they felt their session was beneficial.

Participants

A convenience sample was employed of current patients at the IRF, with diagnoses including cerebrovascular accident, spinal cord injury, brain tumor resection, brain injury, and other neurologic, pulmonary, or orthopedic conditions. Participation was voluntary, and medical team clearance was required. From January 6, 2022, to April 9, 2022, 55 patients self-enrolled and completed 120 weekend sessions.

Procedure

**Figure 2:**
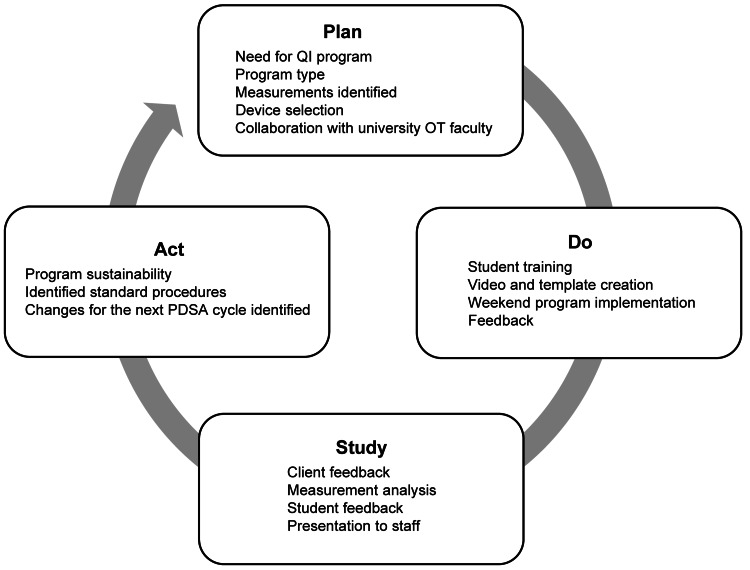
Plan-Do-Study-Act (PDSA) cycle for the weekend robotics program overview Credit: Used with permission from Barrow Neurological Institute, Phoenix, Arizona. OT, occupational therapy; QI, quality improvement

The PDSA cycle guided the program’s iterative development (Figure 2), aligning with the IRF’s quality improvement standards and knowledge transfer principles [11,15,17,24,25]. Documentation was completed in the electronic medical record system using the SOAP (Subjective, Objective, Assessment, Plan) note format and included outcome measures.

Plan Phase

Staff identified patient demand for weekend therapy via satisfaction surveys and planned a student-assisted robotics program. Goals included increased therapy dosage and patient satisfaction; logistics covered device selection and data collection protocols.

Do Phase

From January to April 2022, patients received one- to two-hour-long weekend sessions (Saturday and/or Sunday) using devices including Myomo MyoPro (Myomo, Inc., Burlington, MA, USA), Tyromotion Diego (Tyromotion GmbH, Graz, Austria), Fourier Intelligence ArmMotus M2 (Fourier Intelligence, Shanghai, China), Bioness Integrated Therapy System (Bioness Medical, Inc., Valencia, CA, USA), Bionik Laboratory’s InMotion (Bionik Laboratories Corp., Toronto, Canada), Oculus Quest 2 (Reality Labs/Meta Platforms, Inc., Menlo Park, CA, USA), Microsoft HoloLens 2 (Microsoft Corporation, Redmond, WA, USA), and Neofect Smart Glove (Neofect Inc., Seongnam, South Korea), chosen for their potential to enhance upper extremity function [13,14]. Devices were selected based on individual patient presentation, functional goals, and therapeutic appropriateness, as determined by the supervising licensed occupational therapist. Students worked under direct supervision, with a student-to-therapist ratio of 2-3:1. Device allocation was guided by the therapist's clinical reasoning, incorporating factors such as diagnosis, level of motor impairment (e.g., range of motion), patient tolerance, functional goals, and real-time device availability, rather than a fixed assignment protocol.

Study Phase

Data from 120 sessions were analyzed to assess trends in functional outcomes, safety, effort, and satisfaction quotes, with performance measured against planning goals.

Act Phase

Findings informed refinements, such as streamlined measures and simplified student training for the next cycle, and leadership approved program continuation. This iterative process ensured systematic testing and adaptation to support sustainability and scalability.

## Results

Study phase analysis

During the study phase of the PDSA cycle, researchers evaluated the weekend robotics program’s effectiveness against its objectives: increasing therapy dosage, improving functional outcomes, and enhancing patient satisfaction. From January 6 to April 9, 2022, the program provided 120 hour-long sessions to 55 patients at Barrow Neurological Institute’s IRF. Patients with diagnoses of cerebrovascular accident (22, 40%), spinal cord injury (10, 18%), brain injury (8, 15%), and other neurologic, pulmonary, or orthopedic conditions (15, 28%) participated voluntarily in one or two sessions per weekend. OT students, supervised by licensed therapists, delivered the interventions using Myomo MyoPro, Tyromotion Diego, and Neofect Smart Glove devices.

From the 120 sessions, 10 complete datasets were selected through simple random sampling for outcome analysis to assess the utility and exploratory trends in functional outcomes. Shoulder flexion active range of motion, a primary functional outcome, showed a positive trend in 6 of 10 patients, with an average gain of 12° (range: 3°-22°; e.g., patient 5: 118° to 140°, patient 10: 165° to 180°). Other measures, such as heart rate (mean change: -2 bpm, range: -25 to +35 bpm), oxygen saturation (SpO₂; mean change: 0%, range: -3% to +3%), Borg Rating of Perceived Exertion Scale (mean change: +1.5, range: -2 to +5), and visual analog scale for mood (mean change: +0.2, range: -2 to +2), exhibited minimal shifts, suggesting stable physiological and subjective responses (Tables 1-3).

**Table 1:** Biometric measurement results for 10 patients before and after participation in a student-assisted robotics rehabilitation program NT: not tested

Patient	Heart Rate, bpm		Oxygen Saturation, %			
	Before	After	Before			After
1	96	75		96	99	
2	110	100		98	98	
3	69	65		91	93	
4	117	91		NT	NT	
5	91	100		99	96	
6	63	69		95	94	
7	88	96		NT	NT	
8	61	96		96	96	
9	78	74		98	97	
10	74	71		98	95	

**Table 2:** Perception measurements for 10 patients before and after participation in a student-assisted robotics rehabilitation program NT: not tested

Patient	Borg Rating of Perceived Exertion Scale			Visual Analog Scale for Mood		
	Before	After		Before		After
1	6	7		10		10
2	10	13		10		10
3	NT	NT		7		7
4	11	13		8.5		8.5
5	8	8		10		10
6	6	9		10		10
7	NT	NT		NT		NT
8	7	12		9		10
9	10	8		6		8
10	8	11		10		10

**Table 3:** Physical measurements for 10 patients before and after participation in a student-assisted robotics rehabilitation program NT: not tested

Patient		Left Shoulder Flexion Range of Motion		
		Before	After	
1	155			160
2	12			20
3	78			85
4	NT			NT
5	118			140
6	115			112
7	NT			NT
8	125			112
9	142			145
10	165			180

However, incomplete data in 2 (20%) of the 10 sampled entries (e.g., missing SpO₂ for patients 4 and 7) indicated collection challenges, likely due to the complexity of tracking five measures simultaneously.

Patient self-reported satisfaction was high, with 98% of respondents (82 of 84 post-session responses recorded) affirming the sessions’ benefit when asked posttreatment. This feedback, collected via informal verbal responses, highlighted perceived value and engagement. The program’s 120 additional therapy hours - the equivalent of 24 full weekdays of CMS-mandated therapy - demonstrated a significant dosage increase without impacting weekday staff productivity. This comparison reflects time volume only and does not imply equivalence in treatment mix, interdisciplinary composition, or therapeutic intensity.

Direct quotes from patients that underscore the program’s appeal and perceived therapeutic effect are presented in Table 4.

**Table 4:** Direct quotes from patients underscored the program’s appeal and perceived therapeutic impact

Category	Participant Quotes
Positive experience or enjoyment	“I was anxious before we started, but I had a great time!” “You made my day.”
Perceived benefits	“This is the right kind of burn; I haven’t felt that in a long time.”
Motivation for therapy	“I notice it works everything in my shoulder that needs to be worked.” “I like this; it’s a lot of fun.”

Limitations

This project provided critical insights into the practical challenges of implementing a robotics therapy program. By embracing transparency, we identified several areas for improvement that will enhance future iterations. For instance, addressing the gaps in data collection and refining measurement tools are pivotal steps forward (Table 5).

**Table 5:** Challenges and lessons of the student-assisted robotics rehabilitation program

Challenges	Lessons Learned
Implement required vs. optional outcome measures; Incomplete data collection due to the complexity of measurements; Difficulty identifying validated functional outcome measures	Simplify data collection by focusing on fewer measures; Develop structured documentation templates; Establish predefined procedures for handling missing data
Limited sample size and convenience sampling	Expand recruitment strategies to include diverse participants
Errors in data logging due to a lack of streamlined tools	Develop and use a standardized data collection protocol: Likert-scale measures
No control group for comparative analysis	Include a control group in future study designs

Also, there may be other important outcome measures that were not captured, such as functional ability and standardized test scores. In addition, the use of a convenience sample may not be generalizable to other rehabilitation programs. The potential for selection bias is also present because clients elected to participate. Additional limitations include the use of an informal satisfaction assessment, which is subject to response bias, and the reliance on impairment-level measures rather than validated functional scales. The functional improvements observed in the small subsample should be interpreted as preliminary, exploratory trends rather than evidence of clinical effectiveness. The lack of validated functional outcome measures limits the clinical interpretability of our findings, and the clinical heterogeneity of the sample, which included neurologic, pulmonary, and orthopedic diagnoses with differing recovery trajectories, further limits the interpretation of pooled outcomes. Additionally, no inferential statistical analyses were conducted. Therefore, reported trends should not be interpreted as statistically significant effects.

A benefit of using the PDSA cycle as a quality improvement format is the opportunity to address limitations in the next cycle [21].

## Discussion

This PDSA-driven quality improvement initiative successfully demonstrated the feasibility, safety, and high patient acceptability of a sustainable supplemental weekend robotics program that increased therapy dosage and patient satisfaction in an IRF setting [2,4,5,17,24]. The principal findings of the implementation study relate to operational success, program sustainability, and patient satisfaction, with exploratory trends suggesting potential functional benefits that warrant further investigation in subsequent PDSA cycles. Learning from incomplete data and logistical hurdles reveals that research, though messy, yields valuable insights for healthcare improvement [17]. Transparent reporting of successes and challenges fosters confidence in adapting similar efforts across diverse settings, offering a starting point for providers eager to engage in research through quality improvement. Successful collaboration between academic programs and clinical environments highlights the potential of such partnerships to drive innovation without straining resources [25,26]. This project demonstrates that quality improvement frameworks, like PDSA, can refine care delivery, paving the way for sustainable, patient-centered advancements in rehabilitation [17].

Our weekend robotics program exemplifies the practical application of knowledge transfer principles through the implementation of a therapy program guided by the quality improvement process of the PDSA cycle [15,16]. Despite the challenges encountered and the lack of statistical significance, we believe that the hands-on experience offered to students, and the provision of additional therapy to patients without incurring additional costs to the work unit, underscore the program’s success. Establishing partnerships between clinical sites and academic programs for effectiveness studies represents a promising avenue to address the scarcity of knowledge transfer literature [26]. Future PDSA cycles can strengthen methodological rigor by incorporating standardized functional outcome measures (e.g., Action Research Arm Test, Box and Block Test, or other upper-extremity functional scales), implementing structured satisfaction instruments (e.g., Likert-scale measures), and establishing standardized protocols for data collection, including competency-based measurement training, structured documentation templates, and predefined procedures for handling missing data. These improvements will enhance the clinical interpretability of outcomes and support reproducibility across institutions.

We have demonstrated an approach to knowledge transfer that addresses problem identification (the need to increase therapy interventions), context and barriers (staffing and costs), collaboration (OT program), interventions (weekend robotics program), action, process (education, training, and videos), adjustments (training and staffing), and healthcare outcomes (identifying any changes in scores) [15,17,20,27]. OT practitioners can follow a knowledge transfer or quality improvement framework to establish and evaluate programs, as well as share outcomes with other clinicians. Overall, we emphasize the practical application of knowledge transfer in OT with the implementation of this program on a small scale, to show that it is manageable at this scale and could work for other departments. OT practitioners can use knowledge transfer and existing quality improvement frameworks to enhance the delivery of rehabilitation services and improve patient outcomes [25,26].

## Conclusions

This quality improvement program demonstrates the application of knowledge transfer principles. Our PDSA program serves as a map for healthcare providers to engage in knowledge transfer and quality improvement projects. The hands-on experience offered to students and the provision of additional therapy to patients highlighted the program’s success, providing a replicable quality improvement model for IRFs that does not add incremental costs to the work unit. Future research should include valid Likert-scale measures and explore student outcomes, OT practitioner outcomes, and quality-of-life measures to strengthen the evidence and support wider adoption of PDSA quality initiatives by healthcare providers.
